# Supplementary material for: Androgen receptor is a determinant of melanoma targeted drug resistance
Source: Nat Commun. 2023 Oct 14;14:6498. doi: 10.1038/s41467-023-42239-w (PMC10576812; doi:10.1038/s41467-023-42239-w)
Supplement: Supplementary file 1 — Supplementary figures [file 41467_2023_42239_MOESM1_ESM.pdf]

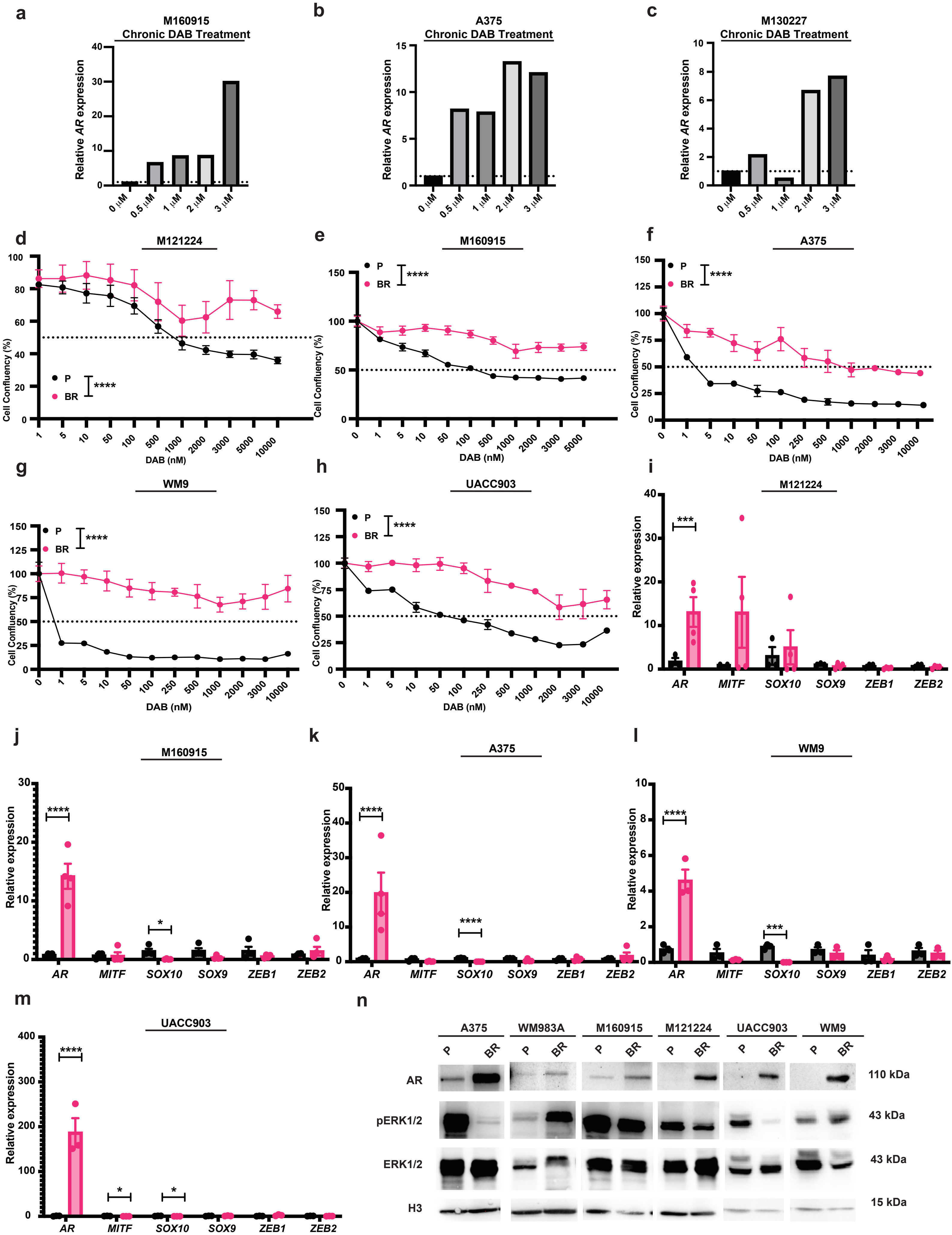
a
M160915
Chronic DAB Treatment
Relative AR expression
0 µM
0.5 µM
1 µM
2 µM
3 µM
b
A375
Chronic DAB Treatment
c
M130227
Chronic DAB Treatment
d
M121224
e
M160915
f
A375
g
WM9
h
UACC903
P
BR
****
Cell Confluency (%)
DAB (nM)
i
M121224
j
M160915
k
A375
l
WM9
m
UACC903
Relative expression
AR
MITF
SOX10
SOX9
ZEB1
ZEB2
***
*
n
A375
WM983A
M160915
M121224
UACC903
WM9
pERK1/2
ERK1/2
H3
110 kDa
43 kDa
15 kDa

Supplementary Fig. 1. BRAFi treatment of melanoma cells results in increased AR expression a-c) *AR* RT-qPCR analysis in additional primary or established human melanoma cell lines besides those analyzed in Fig. 1a. M160915 (a), A375 (b) and M130227 cells (c). Cells were cultured with multistep weekly increases of the BRAF inhibitor Dabrafenib (DAB, 0.5, 1, 2, and 3 μM) and collected at the end of each treatment week. *RPLP0* was used as internal normalization. Related to Fig. 1a.

d-h) Emergence of BRAFi-resistant cells after DAB treatment, assessed via proliferation live-cell imaging assays (IncuCyte). Dose-response curves at various doses upon DAB treatment in A375 (d), M160915 (e), M121224 (f), WM9 (g), and UACC903 (h) cells. Mean ± SD. n(cultures) = 3, two-tailed Log-rank test, (M160915 P - M160915 BR) ****p< 0.0001; (A375 P - A375 BR) ****p< 0.0001;(M121224 P - M121224 BR) ****p< 0.0001; (UACC903 P - UACC903 BR) ****p< 0.0001; (WM9 P - WM9 BR) ****p< 0.0001. Related to Fig. 1a.

i-m) Relative expression of the indicated genes in BRAFi-resistant (BR) primary (B) and established melanoma cell lines (C) versus parental cells (P). Shown are individual bar plots from the heatmap shown Fig. 1d. RT-qPCR results are expressed in arbitrary units relative to untreated controls, after *RPLP0* normalization. Mean ± SEM. n(biological replicates) =3-4, two-tailed t-tests, ***p<0.001, ****p<0.0001. Related to Fig. 1d.

n) Immunoblot (WB) of ERK1/2, phospho-ERK1/2 and AR in A375, WM983A, M160915, M121224, UACC903, WM9 melanoma parental versus BR cells. Histone H3 was used as a loading control. Related to Fig. 1.

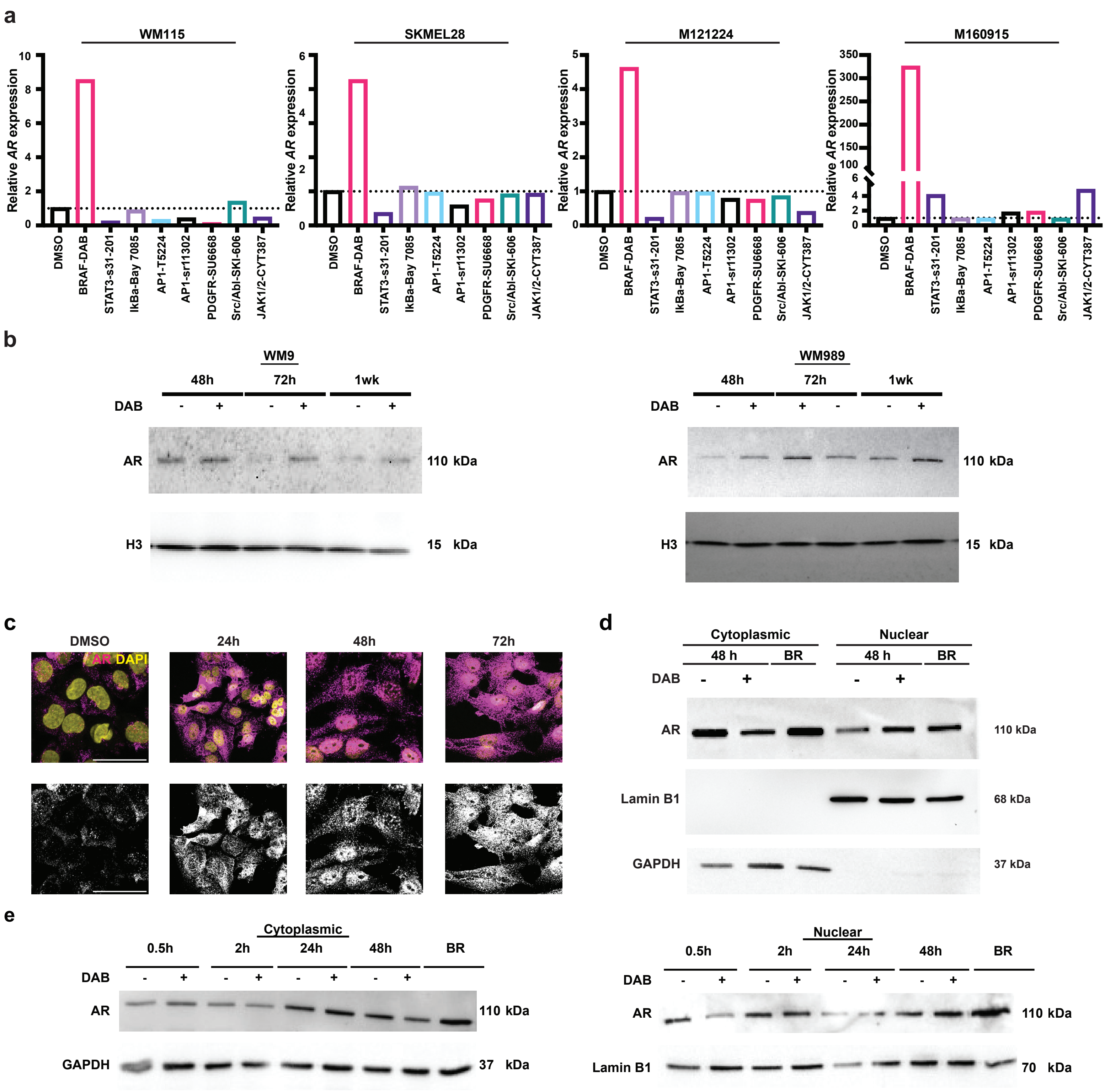
a
WM115
SKMEL28
M121224
M160915
Relative AR expression
DMSO
BRAF-DAB
STAT3-s31-201
IkBa-Bay 7085
AP1-T5224
AP1-sr11302
PDGFR-SU6668
Src/Abl-SKI-606
JAK1/2-CYT387
b
WM9
WM989
48h
72h
1wk
DAB
AR
H3
110 kDa
15 kDa
c
DMSO
24h
48h
72h
AR DAPI
d
Cytoplasmic
Nuclear
48 h
BR
DAB
AR
Lamin B1
GAPDH
110 kDa
68 kDa
37 kDa
e
Cytoplasmic
Nuclear
0.5h
2h
24h
48h
BR
DAB
AR
GAPDH
Lamin B1
110 kDa
37 kDa
70 kDa

Supplementary Fig. 2. Acute BRAFi treatment of melanoma cells results in increased AR expression and nuclear localization.

a) RT-qPCR analysis of *AR* upon BRAFi treatment or inhibitors of NF-kB, STAT3, and AP-1 in WM115, SKMEL28, M121224, and M160915 cell lines. *RPLP0* was used as internal normalization. n(biological replicates)= 2. Mean, Related to Fig. 2a.
b) AR and Histone3 (H3) WBs in WM9 and WM989 cell lines treated with DAB (0.5 μM) versus DMSO control at the indicated time points. Related to Fig. 2b-d. The experiment was performed independently twice in multiple melanoma lines with similar results.
c) Representative images of AR expression in A375 melanoma cells treated with DAB (0.5 μM) versus DMSO control at the indicated time points. Color scale: yellow, DAPI; magenta, AR. Scale bar: 10 μm. Related to Fig. 2f.
d) AR, LaminB1 and GAPDH WBs in A375 melanoma cells treated for, 48 hours with DAB (0.5 μM) versus DMSO control cells. Cytoplasmic and nuclear cell fractions are shown, with as a nuclear loading control and GAPDH as a cytoplasmic loading control. Related to Fig. 2g-h.
e) AR, LaminB1 and GAPDH WBs in A375 melanoma treated for 0.5-, 2-, 24-, and 48 hours with DAB (0.5 μM) versus DMSO control cells. Cytoplasmic and nuclear cell fractions are shown, with LaminB1 as a nuclear loading control and GAPDH as a cytoplasmic loading control. Related to Fig. 2g-h.

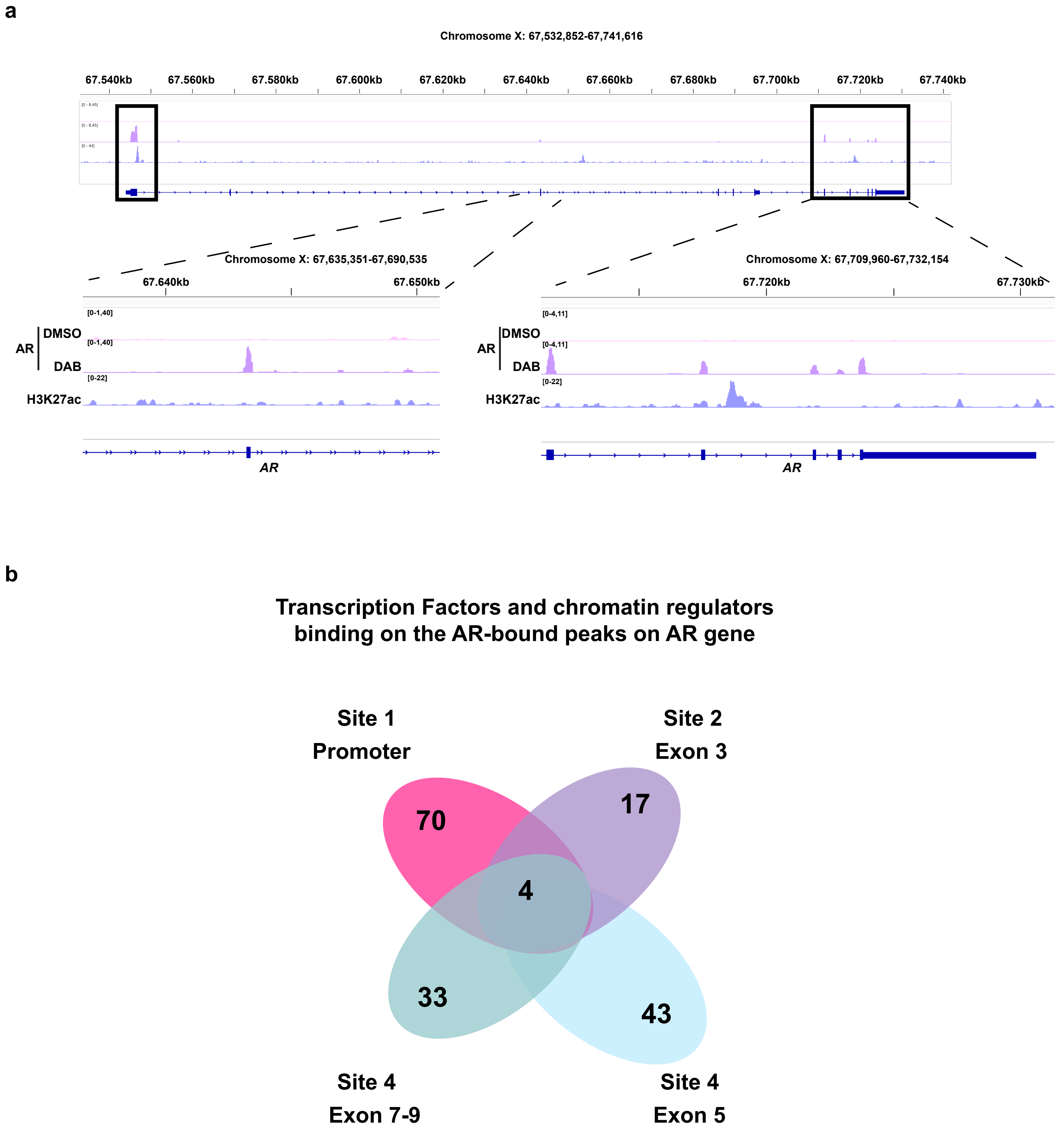

Supplementary Fig. 3. BRAFi treatment induces AR binding on the regulatory elements of AR gene.

a) Illustration of AR binding peaks in A375 cells to *AR* promoter displayed using the integrative genomic viewer software (IGV). AR binding peaks in DAB-treated (deep purple), DMSO control (cyan), and H3K27ac (light purple) peaks derived from Joung et al., Nature, 2017 [54] were used to map histone modifications overlapping with AR binding regions. Related to Fig. 3d.

b) Venn diagram illustrating common and differential transcription factors and chromatin regulators predicted to bind to the same AR binding peaks as in DAB-treated in the *AR* gene. Related to Fig. 3e.

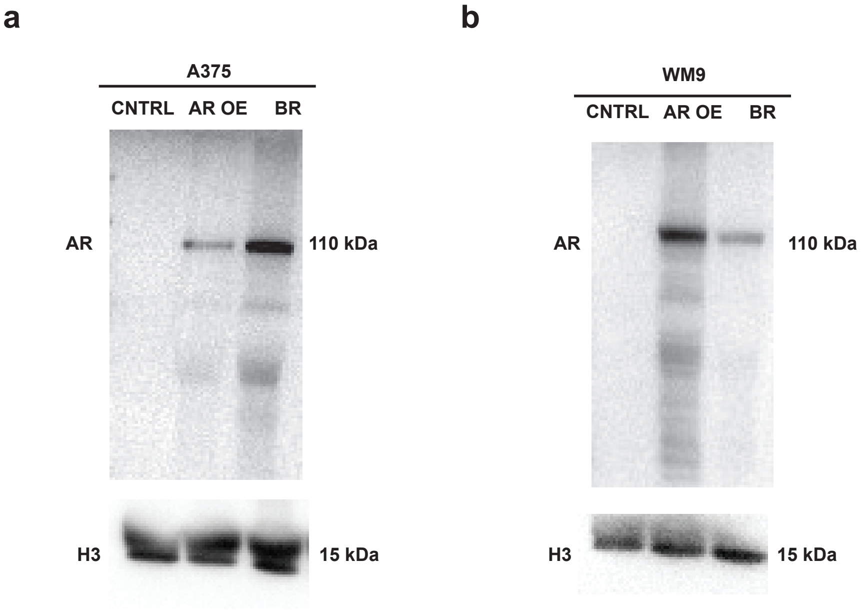

Supplementary Figure 4: Validation of AR overexpression in melanoma cell lines.
a-b) Immunoblotting of AR expression in A375 (a) and WM9 (b) melanoma cell lines stably infected with an AR overexpressing lentivirus (AR OE) versus LacZ expressing control (CNTRL) relative to the BRAFi-resistant A375BR and WM9BR melanoma cells. Histone3 (H3) and GAPDH were used as an equal loading control. Related to Fig. 4.

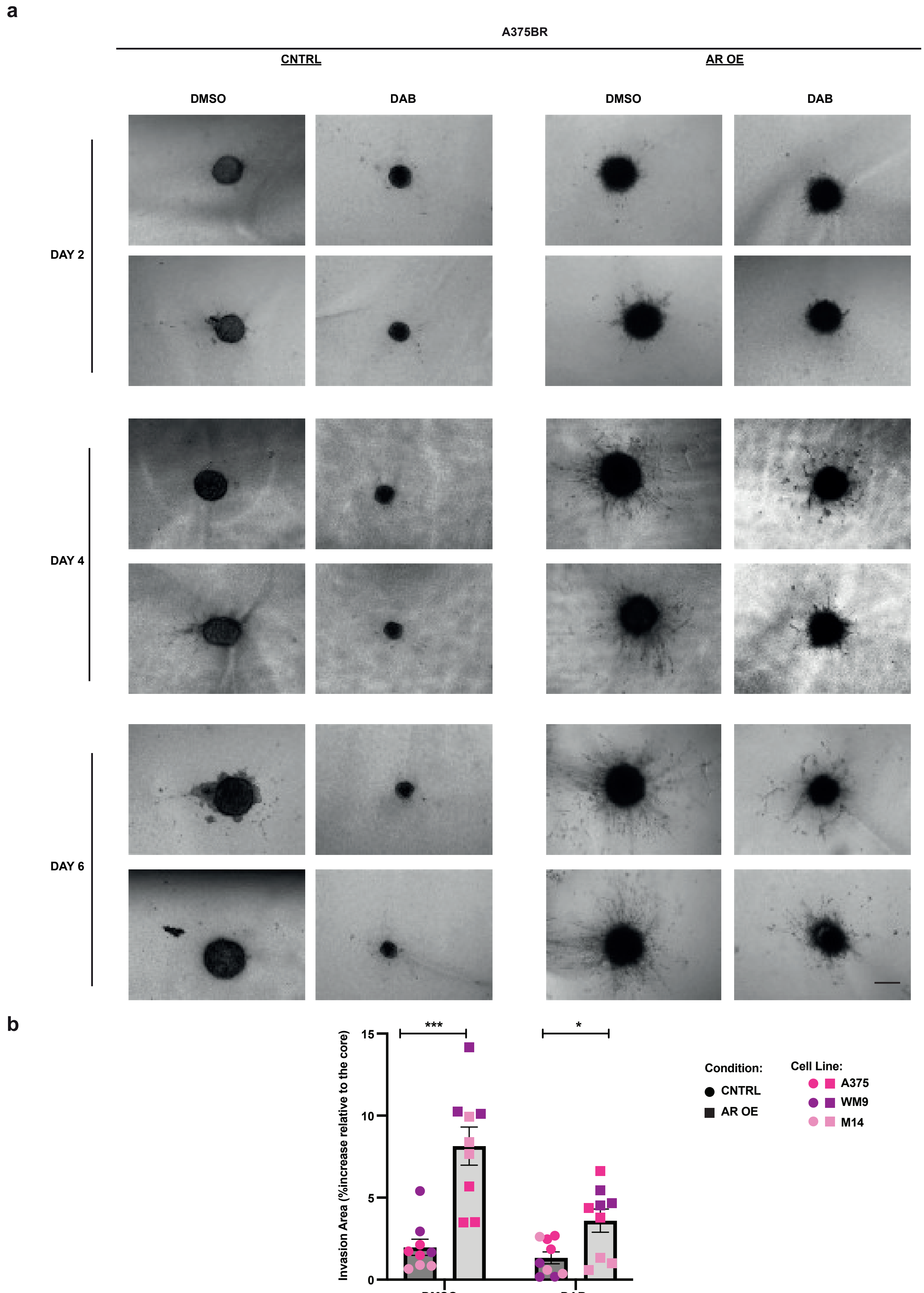

Supplementary Figure 5: Increased AR levels drive invasive properties of melanoma cells.

a) Brightfield images of multicellular spheroid invasion into the 3D collagen lattices by A375 melanoma cells on day 2, 4 and 6. Scale bars: 400µm.
b) Quantification of invasion area of A375, WM9 and M14 spheroids 4 days (96h) post-embedding. Mean ± SD. N(dishes/cell line) = 3, two-tailed unpaired t-test, ****p< 0.0001, *p<0.05. Summary per condition: DMSO AR OE - CNTRL p=0.0002; DAB AR OE - CNTRL p=0.00114.

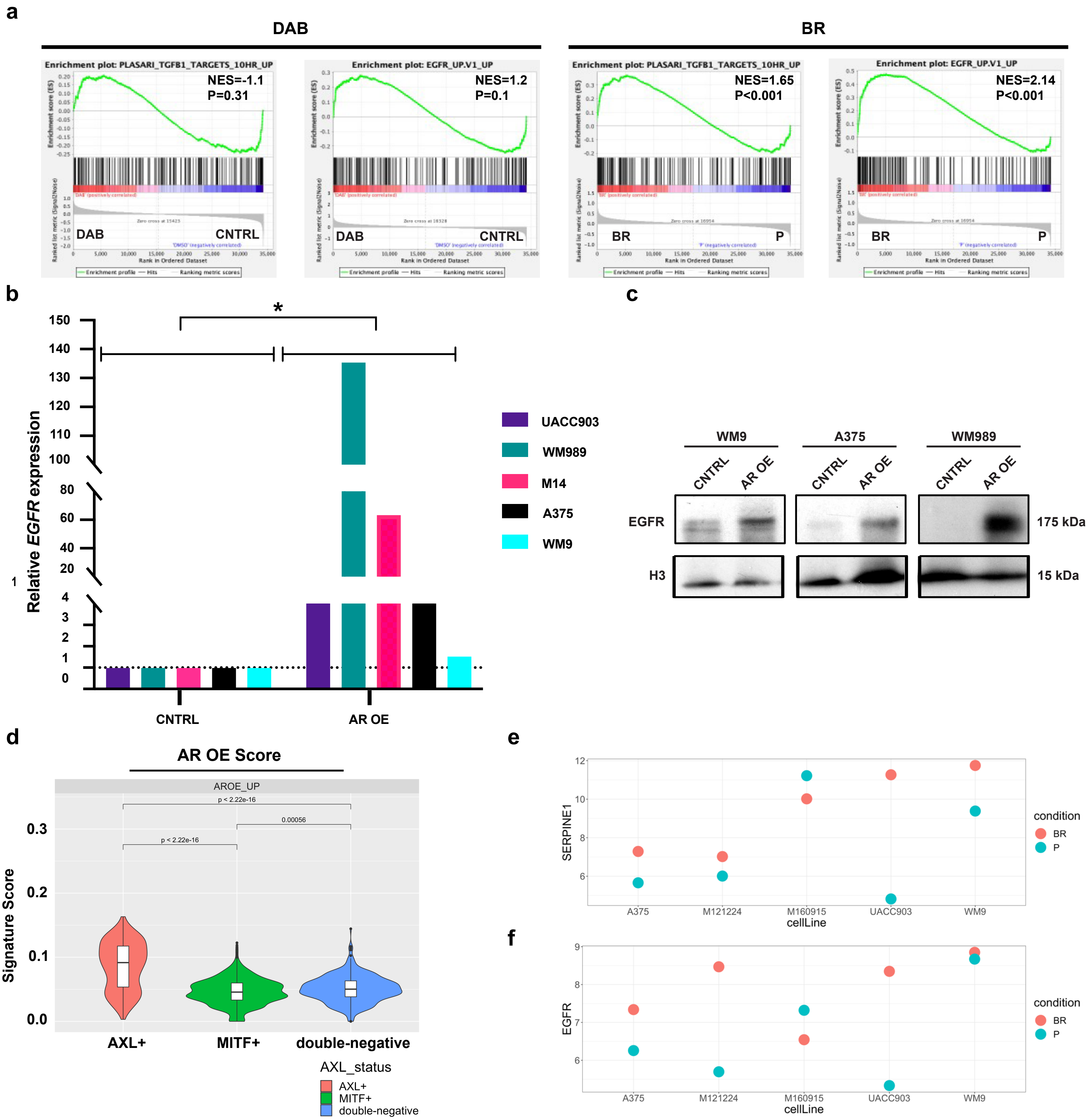

Supplementary Figure 6: Increased AR levels drive expression of EGFR and TGF-ß targets.

a) GSEA of gene signatures related to EGFR (EGFR UP) and TGF-ß (TGFB1 targets) signaling. Genes are ranked by signal-to-noise ratio in DAB-treated melanoma cells versus DMSO-treated melanoma cells (left) or in BRAFi-resistant melanoma cells versus parental melanoma cells (right). The position of individual genes is indicated by black vertical bars; the enrichment pattern is in green.

b) RT-qPCR analysis of *EGFR* expression in WM9, A375, M14, UACC903 and WM989 melanoma cells stably infected with AR versus LacZ expressing (control) lentiviruses. EGFR expression (arbitrary units) relative to control conditions, after *RPLP0* normalization. N =5. Related to Fig. 6h.

c) Immunoblotting of EGFR in WM9, A375 and WM989 melanoma cell lines stably infected with an AR overexpressing lentivirus or LacZ expressing control. Histone H3 was used as an equal loading control. EGFR was assessed on the same membrane as used in Fig. 6C. Related to Fig. 6h.The experiment was performed independently twice with similar results.

d) AR OE signature score in cell subpopulations identified by single-cell RNA-seq analysis of treatment-naïve melanoma tumors from 33. A gene signature of 19 upregulated and 39 downregulated genes (p-value<0.01, absolute FC>1) in the AR overexpressing versus control melanoma cells was established (for a list of genes see Supplementary Table 3) and used to calculate scores of AR activity, using AUCell, in the scRNA-seq profiles of previously defined populations of the AXL+, MITF+ or double-negative tumor subpopulations. Violin plots show individual cell score distribution within each cell population.In the box plots, the center line shows the median, the box edges delineate 25th and 75th percentiles. The significance of differences in mean score values between invasive versus naïve cell populations was calculated by two-tailed Welch's t-test . p-values are as follows: AXL+ - MITF+ $p=2.22x10^{-22}$; AXL+ - double-negative $p=2.22x10^{-22}$; MITF+ double-negative p=-0.00056 .

e-f) Levels of *EGFR* (e) and *SERPINE1* (f) expression from the transcriptional profiles of BR versus parental melanoma cells. Shown are the expression levels of *EGFR* (e) and *SERPINE1* (f) in A375, WM983A, M160915, M121224, UACC903, WM9 melanoma parental (blue dots) versus BR (red dots) melanoma cells.

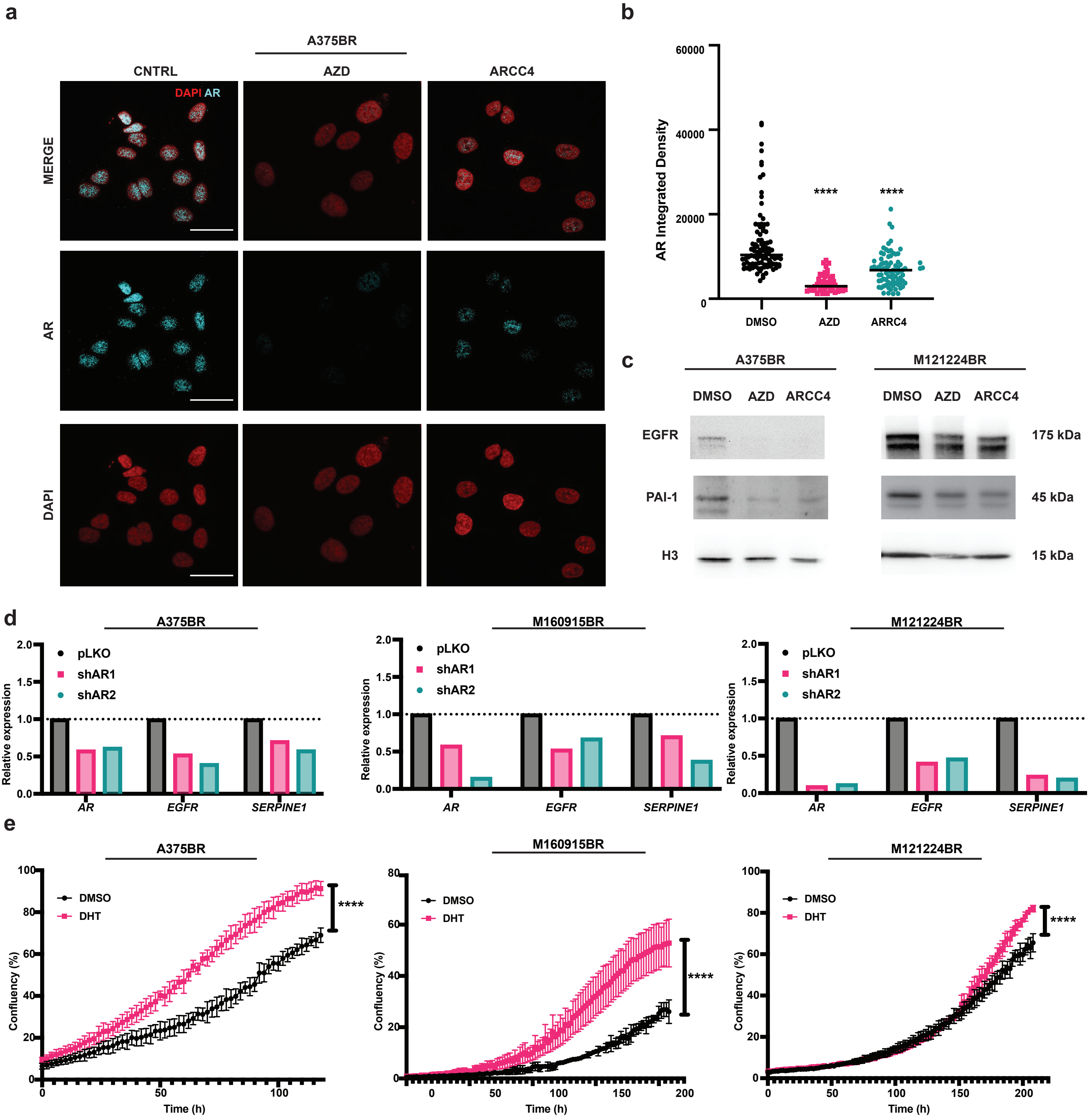

Supplementary Figure 7: AR inhibition decreases AR, EGFR, and PAI-1 protein expression in BRAFi-resisant melanoma cells. a-b) Immunofluorescence analysis of BRAF inhibitor-resistant melanoma cells (A375BR) treated with the AR inhibitor AZD3514 (10 µM) or ARCC4 (1 µM) versus DMSO control for 48 hours with anti-AR antibodies with DAPI staining for nuclei visualization. Shown are representative images (a) and quantification (b) of AR signal intensity in arbitrary units (AU) per individual cells (dots) together with a mean, examining >100 cells per sample, unpaired t-test, **** $p<0.0001$. Color scale: red, DAPI; cyan, AR. Scale bar: 40µm. Related to Fig. 8a.

c) Immunoblot analysis of EGFR and PAI-1 expression in A375BR and M121224BR cells treated with AZD3514 or ARCC4 versus DMSO control. Histone3 (H3) was used as an equal loading control. Related to Fig. 8a-b.
d) RT-qPCR analysis of AR, EGFR and SERPINE1 expression in A375BR, M121224BR and M160915BR cells following infection with two AR-silencing lentiviruses versus empty control.
e) Proliferation live-cell imaging assays (IncuCyte) of the A375BR, M121224BR and M160915BR continuously grown in the presence of Dabrafenib (as specified in Methods) plus/minus treatment with DHT (10 nM) or DMSO control. Cells were plated in triplicate wells in 96-well plates followed by cell density measurements (four images per well every 2 h for >100 h). cultures, N = 3. Mean ± SD; Pearson r correlation test, ****, $p< 0.0001$.

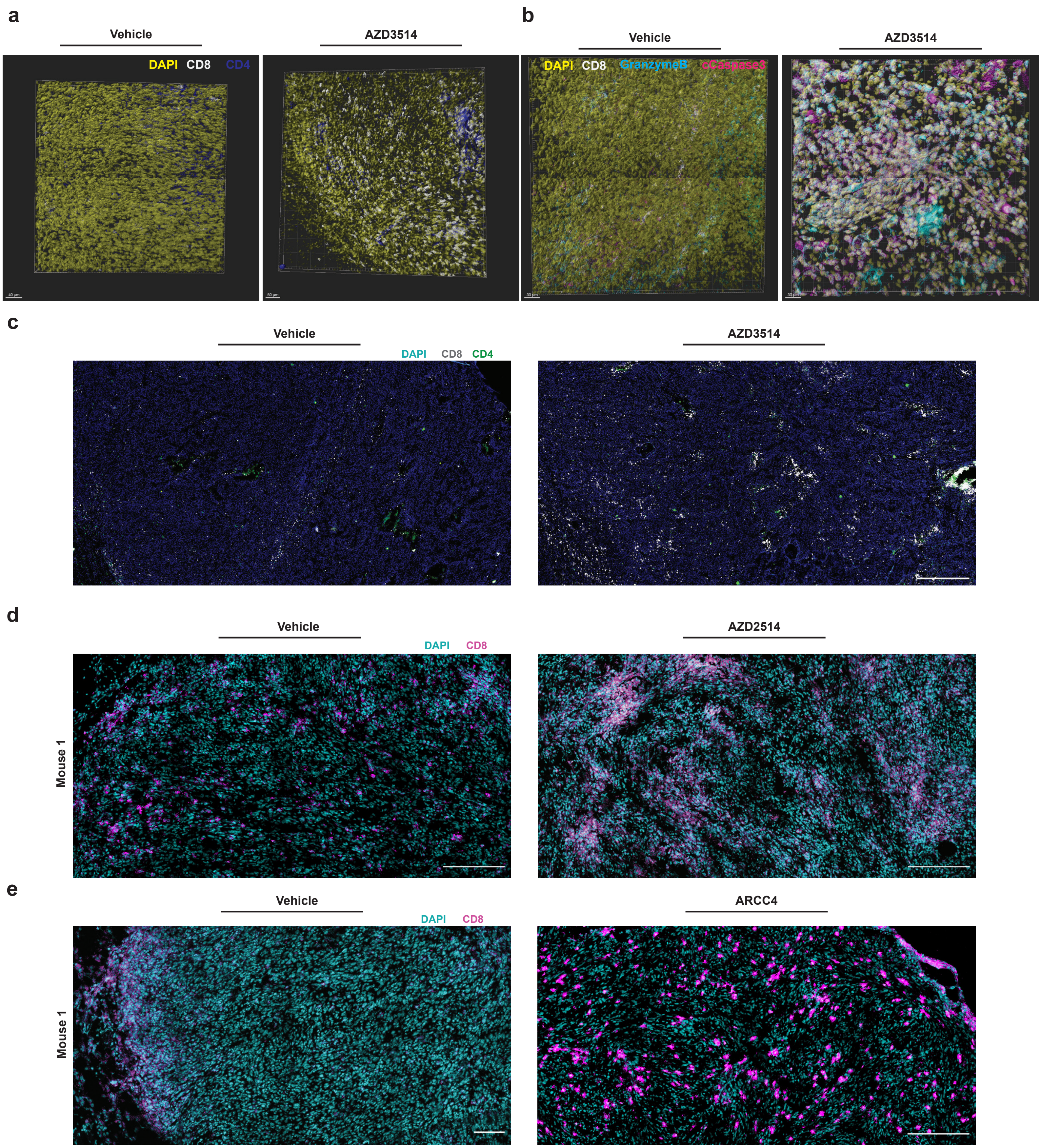
a
Vehicle
AZD3514
DAPI CD8 CD4
b
Vehicle
AZD3514
DAPI CD8 GranzymeB cCaspase3
c
Vehicle
AZD3514
DAPI CD8 CD4
d
Vehicle
AZD2514
DAPI CD8
Mouse 1
e
Vehicle
ARCC4
DAPI CD8
Mouse 1

Supplementary Figure 8. Pharmacological AR targeting alters CD8 T cell infiltration *in vivo*.

a) IF with anti-CD4+ and anti-CD8+ antibodies and 3D reconstruction analysis by Imaris Software of thickX sections (40 µM) of tumors from immunocompetent mice injected with YUMM1.7BR cells and treated with AZD3514 versus DMSO for 14 days as in (Fig. 9f). Color scale: yellow, DAPI; white, CD8, blue, CD4. Scale bar: 30 µm. Related to Fig. 9i.
b) IF with antibodies against cleaved caspase 3, granzyme B and the CD8 T cell maker and 3D reconstruction analysis by Imaris Software of thick sections (40 µM) of tumors from immunocompetent mice injected with YUMM1.7BR cells and treated with AZD3514 versus DMSO for 14 Days as in (Fig. 9f). Color scale: yellow, DAPI; grey, CD8; cyan, granzyme B; magenta, cleaved caspase 3. Scale bar: 30 µm. Related to Fig. 9j.
c) Representative immunofluorescence images of the excised YUMM1.7BR tumors treated with AZD3514 versus DMSO vehicle for 14 days. Shown are representative low-magnification images of the CD8+ T cell areas. Color scale: blue, DAPI; green, CD4+; white, anti-CD8+ antibodies. Scale Bar: 250 µm. Related to Fig. 9l.

d-e) Immunofluorescence analysis of the excised YUMM1.7BR tumors subjected to a single treatment with AZD3514 (10 µM) (d) or ARCC4 (1 µM) (e). Shown are representative low magnification images of the CD8+ T cell areas. Color scale: cyan, DAPI; magenta, anti-CD8+ antibodies. Scale Bar: 100 µm. Related to Fig. 9m.

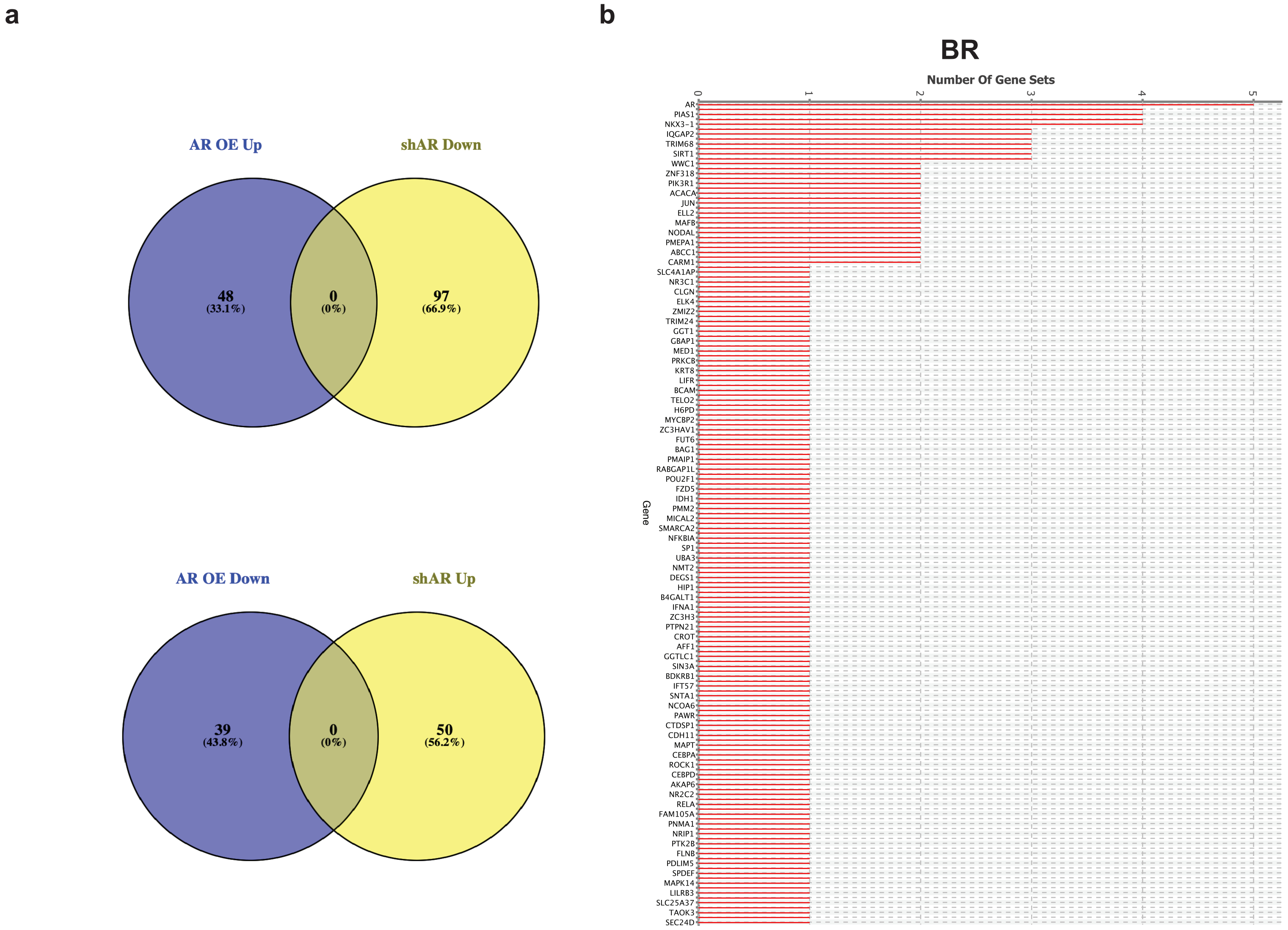

Supplementary Figure 9: Comparative analysis of AR regulated genes in the transcriptomic profiles of melanoma cells with AR silencing, overexpression, BRAFi treatment and BRAF resistance.

a) Venn diagrams illustrating common and differentially expressed genes in multiple melanoma cell lines plus/minus AR gene silencing as previously reported 9 versus those in melanoma cells plus/minus AR overexpression (Supplementary Table 3). Number of significantly modulated genes that were oppositely modulated in the two conditions (FC>2; p-value<0.05) is indicated.

b) Leading edge analysis of 17 different gene signatures measuring AR activity in prostate cells derived from the literature (GSEA MSigDB and 46) for GSEA in melanoma profiles under control conditions versus 48 hours of DAB treatment (left), AR overexpression (center) and BRAFi resistance (right). Red color indicates genes positively enriched in the indicated conditions as a function of AR expression in melanoma cells. A full list of leading edge genes and the AR gene signatures is provided in the Supplementary Table 1.

SUPPLEMENTARY TABLES

Supplementary Table 1: List of differentially expressed genes and gene ontology terms enriched in the parental and BRAF-resistant melanoma cells.
Supplementary Table 2: List of differentially expressed genes and gene ontology terms enriched in control and AR overexpressing cells plus/minus Dabrafenib treatment.
Supplementary Table 3: List of differentially expressed genes and GSEA terms enriched in AR overexpressing cells under basal conditions.
Supplementary Table 4: List of cell lines used in the study.
Supplementary Table 5: List of key reagents and resources used in the study.
