# Supplementary material for: Androgen receptor is a determinant of melanoma targeted drug resistance
Source: Nat Commun. 2023 Oct 14;14:6498. doi: 10.1038/s41467-023-42239-w (PMC10576812; doi:10.1038/s41467-023-42239-w)
Supplement: Supplementary file 3 — Description of Additional Supplementary Files [file 41467_2023_42239_MOESM3_ESM.pdf]

## Description of Additional Supplementary Files

Supplementary Data 1
Description: List of differentially expressed genes and gene ontology terms enriched in the parental and BRAF-resistant melanoma cells.

Supplementary Data 2
Description: List of differentially expressed genes and gene ontology terms enriched in the control and AR overexpressing cells plus/minus Dabrafenib treatment.

Supplementary Data 3
Description: List of differentially expressed genes and GSEA terms enriched in AR overexpressing cells under basal conditions.

Supplementary Data 4
Description: List of cell lines used in the study.

Supplementary Data 5
Description: List of key reagents and resources used in the study.
