# Supplementary material for: Androgen receptor is a determinant of melanoma targeted drug resistance
Source: Nat Commun. 2023 Oct 14;14:6498. doi: 10.1038/s41467-023-42239-w (PMC10576812; doi:10.1038/s41467-023-42239-w)
Supplement: Supplementary file 5 — Reporting Summary [file 41467_2023_42239_MOESM5_ESM.pdf]

nature portfolio

Corresponding author(s): Gian Paolo Dotto

Last updated by author(s): Aug 20, 2023

Reporting Summary

Nature Portfolio wishes to improve the reproducibility of the work that we publish. This form provides structure for consistency and transparency in reporting. For further information on Nature Portfolio policies, see our Editorial Policies and the Editorial Policy Checklist.

Statistics

For all statistical analyses, confirm that the following items are present in the figure legend, table legend, main text, or Methods section.

n/a	Confirmed	
☐	☒	The exact sample size (*n*) for each experimental group/condition, given as a discrete number and unit of measurement
☐	☒	A statement on whether measurements were taken from distinct samples or whether the same sample was measured repeatedly
☐	☒	The statistical test(s) used AND whether they are one- or two-sided<br>*Only common tests should be described solely by name; describe more complex techniques in the Methods section.*
☒	☐	A description of all covariates tested
☐	☒	A description of any assumptions or corrections, such as tests of normality and adjustment for multiple comparisons
☐	☒	A full description of the statistical parameters including central tendency (e.g. means) or other basic estimates (e.g. regression coefficient) AND variation (e.g. standard deviation) or associated estimates of uncertainty (e.g. confidence intervals)
☐	☒	For null hypothesis testing, the test statistic (e.g. *F*, *t*, *r*) with confidence intervals, effect sizes, degrees of freedom and *P* value noted<br>*Give P values as exact values whenever suitable.*
☒	☐	For Bayesian analysis, information on the choice of priors and Markov chain Monte Carlo settings
☒	☐	For hierarchical and complex designs, identification of the appropriate level for tests and full reporting of outcomes
☐	☒	Estimates of effect sizes (e.g. Cohen's *d*, Pearson's *r*), indicating how they were calculated

Our web collection on statistics for biologists contains articles on many of the points above.

Software and code

Policy information about availability of computer code

Data collection	All the data used in the manuscript was obtained from publicly available depositories and websites, including GEO and MSigDB. The GEO numbers are specified in Methods section of the manuscript.<br>Light Cycler 480 (Roche) associated software<br>ZEISS LSM880 confocal microscope and NanoZoomer S60 microscope associated softwares<br>Nikon Ndp.View2<br>Fiji/ImageJ<br>Imaris software v9 (Oxford Instruments)
Data analysis	limma 3.56.2<br>AUCell 1.22.0<br>clusterProfiler 4.0<br>Seurat 4.1.1<br>Gene set enrichment analysis GSEA 4.0.1<br>R package ‘survival’ v2.43-3<br>Enrichr<br>Incucyte® ZOOM Software<br>Graph Pad Prism 9 Software<br>ImageJ2 v2.3.0/1.53q<br>Microsoft Excel 365<br>Integrative Genomic Viewer 2.8.13 (MIT/Broad)<br>MACS2

the Burrows-Wheeler Aligner (BWA) 70
PeaksAnnotator (HOMER)
Bowtie2 Version 2.3.0

For manuscripts utilizing custom algorithms or software that are central to the research but not yet described in published literature, software must be made available to editors and reviewers. We strongly encourage code deposition in a community repository (e.g. GitHub). See the Nature Portfolio guidelines for submitting code & software for further information.

Data

Policy information about availability of data

All manuscripts must include a data availability statement. This statement should provide the following information, where applicable:
- Accession codes, unique identifiers, or web links for publicly available datasets
- A description of any restrictions on data availability
- For clinical datasets or third party data, please ensure that the statement adheres to our policy

GSE232690 GSE232693 and GSE199405 have been grouped under SuperSeries GSE232697.
To review GEO accession GSE232697:
Go to https://www.ncbi.nlm.nih.gov/geo/query/acc.cgi?acc=GSE232697
Enter token sberisaixxsxlib into the box

Human research participants

Policy information about studies involving human research participants and Sex and Gender in Research.

Reporting on sex and gender	De-identified melanoma samples and sections from the Live Cell Biobanks of the University Research Priority Program (URPP) "Translational Cancer Research"
Population characteristics	N/A
Recruitment	N/A
Ethics oversight	BASEC-Nr 2017—00494

Note that full information on the approval of the study protocol must also be provided in the manuscript.

Field-specific reporting

Please select the one below that is the best fit for your research. If you are not sure, read the appropriate sections before making your selection.

☒ Life sciences ☐ Behavioural & social sciences ☐ Ecological, evolutionary & environmental sciences

For a reference copy of the document with all sections, see nature.com/documents/nr-reporting-summary-flat.pdf

Life sciences study design

All studies must disclose on these points even when the disclosure is negative.

Sample size	Sample size was determined using power calculation allowing the use of statistical analysis.<br>Statistical significance of differences between experimental groups and controls was assessed by two-tailed unpaired or paired t-test, and one way ANOVA, as indicated in the legends. In all cases, p values < 0.05 were considered as statistically significant.<br>In animal experiments, we used a power calculation to determine the minimum sample size that is needed in order to have sufficient statistical power to detect a treatment effect (20-30% change in tumor growth).
Data exclusions	No data or sample were excluded from the analysis.
Replication	The experiments were independently replicated with multiple cell lines, with technical replicates throughout the study. All attempts were successful. No cell line or data were excluded.
Randomization	In all the experiments, the animals were injected contralaterally with both control and treated cells. In the gavage experiments the animals were randomly assigned to control or treated group.
Blinding	The researchers involved in the study were not blinded during sample obtainment or data analysis. Considering the multiple aspects of the study, complete blinding of the investigators was not possible for the data collection, nor relevant considering the cross-check of the results.<br>In vitro experiments and bioinformatic data analysis were cross checked by the Investigators involved in the study.<br>The in vivo experiments were carried out as a team.

Reporting for specific materials, systems and methods

We require information from authors about some types of materials, experimental systems and methods used in many studies. Here, indicate whether each material, system or method listed is relevant to your study. If you are not sure if a list item applies to your research, read the appropriate section before selecting a response.

Materials & experimental systems

n/a	Involved in the study
☐	☒ Antibodies
☐	☒ Eukaryotic cell lines
☒	☐ Palaeontology and archaeology
☐	☒ Animals and other organisms
☒	☐ Clinical data
☒	☐ Dual use research of concern

Methods

n/a	Involved in the study
☐	☒ ChIP-seq
☒	☐ Flow cytometry
☒	☐ MRI-based neuroimaging

Antibodies

Antibodies used

A full list of primary and secondary antibodies, including catalog numbers, clone names and dilutions, used in the study is provided in Supplementary Table 5.
REAGENT or RESOURCE
Antibodies SOURCE Catalog number RRID Assays and dilution used
Rabbit monoclonal anti-AR (D6F11) Cell Signaling Technology Cat# 5153S AB_10691711 WB (1:1000), IF (1:200)
Mouse Monoclonal Anti-Androgen receptor Antibody, Sino Biologicals Cat# 100272-MM05 (clone #5) NA IF (1:200)
Mouse Monoclonal Anti-p-AR/C Antibody (E-6) Santa Cruz Biotechnology Cat# sc-377546 NA IF (1:200)
Rabbit monoclonal anti-EGF Receptor (D38B1) Cell Signaling Technology Cat# 4267 AB_2246311 WB (1:1000)
Goat polyclonal anti-EGFR Novus Biologicals Cat# AF231 AB_355220 IF(1:200)
Rabbit polyclonal anti-MELANA Sigma-Aldrich Cat# HPA048662 AB_2680485 IF/IHC (1:500)
Mouse monoclonal anti-Melan-A (A103) Santa Cruz Biotechnology Cat# sc-20032 AB_627912 IF/ IHC (1:200)
Rabbit polyclonal anti-Serpin E1/PAI-1 Novus Biologicals Cat# 19773 AB_1642775 WB (1:1000), IF (1:200)
Rabbit polyclonal anti-GAPDH (FL-335) Santa Cruz Biotechnology Cat# sc-25778 AB_10167668 WB (1:10000)
Rabbit monoclonal anti-Histone H3 (D1H2) Cell Signaling Technology Cat# 4499 AB_10544537 WB (1:10000)
Rabbit monoclonal anti-Axl Cell Signaling Technology Cat# 3269 AB_2062562 WB (1:1000)
Mouse Monoclonal anti-granzyme B (#AF1865, R&D systems, 1:500)
Rabbit monoclonal anti-cleaved caspase-3 (#9661, Cell Signaling Technology, 1:500)
Goat polyclonal anti-AXL R&D Cat# AF154 AB_354852 IF (1:500)
Mouse monoclonal anti-MHC I (W6/32) PE Santa Cruz Biotechnology Cat# sc-32235 AB_627934 IF (1:500)
Rat monoclonal CD8α (53–6.7) BD Biosciences Cat# 550281 AB_2275792 IF (1:1000)
Goat anti-rabbit IgG HRP Promega Cat# W401B AB_430833 WB (1:10000)
Donkey anti-mouse IgG Alexa Fluor™ 488 ThermoFisher Cat# A-21202 AB_141607 IF (1:1000)
Donkey anti-rabbit IgG Alexa Fluor™ 568 ThermoFisher Cat# A-10042 AB_2534017 IF (1:1000)
Donkey anti-goat IgG Alexa Fluor™ 647 ThermoFisher Cat# A-21447 "
AB_2535864" IF (1:1000)
Chicken anti-Rat IgG Alexa Fluor™ 647 ThermoFisher Cat# A-21472 AB_2535875 IF (1:1000)
Donkey anti-rabbit IgG Alexa Fluor™ 488 ThermoFisher Cat# A-21206 "
AB_2762833" IF (1:1000)
Donkey anti-mouse IgG Alexa Fluor™ 568 ThermoFisher Cat# A10037 AB_2534013 IF (1:1000)
Chicken anti-Rabbit IgG Alexa Fluor™ 647 ThermoFisher Cat# A-21443 AB_2535861 IF (1:1000)
Donkey anti-goat IgG Alexa Fluor™ 488 ThermoFisher Cat# A-11055 AB_2534102 IF (1:1000)

Validation

All antibodies underwent an application-specific validation by the companies. For most, we also indicate the Research Resource Identifier (https://scicrunch.org/resources), providing a link to the validation and previous published use of each antibody.

Eukaryotic cell lines

Policy information about cell lines and Sex and Gender in Research

Cell line source(s)

A full list of different melanoma cell lines and primary melanoma cells and their characteristics is provided in Supplementary Table S4.
Cell lines RRID Source Age & Sex Disease stage Mutations
WM1552C CVCL_6472 Dr. Meenhard Herlyn, Wistar Instititue, USA 72/Male Primary/RGP BRAF,PTEN,TP53
WM115 CVCL_0040 ATCC, CRL-1676 55/Female VGP BRAF,PTEN
WM983A CVCL_A338 Dr. Meenhard Herlyn, Wistar Instititue, USA 54/Male VGP BRAF,TP53
A375 CVCL_0132 ATCC, CRL-1619 54/Female Met BRAF
SKMEL28 CVCL_0526 ATCC, HTB-72 51/Male Met BRAF,PTEN,TP53,CDK4
M14 John Wayne Cancer Institute 33/Male Met BRAF,TP53
WM9 CVCL_6806 Dr. Meenhard Herlyn, Wistar Instititue, USA M Lymph Met Cut BRAF, CDKKN2A, TERT
WM989 CVCL_0B84 Dr. Meenhard Herlyn, Wistar Instititue, USA F Cut BRAF; TP53; CDKN2A

	UACC903 CVCL_4052 Dr. Meenhard Herlyn, Wistar Instititue, USA 25/M Met Cut BRAF, PTEN<br>M121224 Dr. Reinhard Dummer/ Dr Mitch Levesque, University Zürich 40/M Skin Met BRAFV600E, NRAS Q61K<br>M160915 Dr. Reinhard Dummer/ Dr Mitch Levesque, University Zürich 33/F Nodular NRAS Q61K<br>MM130227 Dr. Reinhard Dummer/ Dr Mitch Levesque, University Zürich 85/F Unknown NRAS Q61K
Authentication	Cell morphology and growth characteristics were monitored throughout the study and compared with the previously published reports. No further authentication of these cell lines was performed.
Mycoplasma contamination	All cell lines were routinely checked for the absence of mycoplasma by HOECHST staining without permeabilization of parallel cultures. Observation at high magnification (630x) showed that all were negative.
Commonly misidentified lines<br>(See ICLAC register)	No commonly misindentified cell strains were used

Animals and other research organisms

Policy information about studies involving animals; ARRIVE guidelines recommended for reporting animal research, and Sex and Gender in Research

Laboratory animals	Mouse: NOD.CB17-Prkdcscid/J (6-8 weeks, males)<br>Mouse : C57BL/6J (6-8 weeks, males)
Wild animals	No wild animals were used.
Reporting on sex	The experiments were performed in male mice.
Field-collected samples	No field collected samples were used in the study.
Ethics oversight	All animal studies were carried out according to Swiss guidelines for the use of laboratory animals, with protocols approved by the University of Lausanne animal care and use committee and the veterinary office of Canton Vaud (animal license No. 1854.4f/1854.5a).

Note that full information on the approval of the study protocol must also be provided in the manuscript.

ChIP-seq

Data deposition

☒ Confirm that both raw and final processed data have been deposited in a public database such as GEO.

☒ Confirm that you have deposited or provided access to graph files (e.g. BED files) for the called peaks.

Data access links<br>*May remain private before publication.*	To review GEO accession GSE232697:<br>Go to https://www.ncbi.nlm.nih.gov/geo/query/acc.cgi?acc=GSE232697<br>Enter token sberisaixxslib into the box
Files in database submission	BEDGRAPH
Genome browser session<br>(e.g. UCSC)	Bedgraphs are provided in the GEO to allow visualization in IGV.

Methodology

Replicates

All reads were pair-ended in each condition. The total number of reads, unque number of reads and the length of reads were as follows:

Sample Reads	Clean_reads	Mapped	Unique_mapped
LacZ_Ct_Inp pair	14510254	10960884(75.54%)	8440903(77.01%)
LacZ_Ct_Inp read1	14510254	14509918(100.00%)	10196235(70.27%)
LacZ_Ct_Inp read2	14510254	14509931(100.00%)	10014563(69.02%)
ARoe_Ct_AR pair	17096835	10712588(62.66%)	8384631(78.27%)
ARoe_Ct_AR read1	17096835	16630356(97.27%)	9731394(58.52%)
ARoe_Ct_AR read2	17096835	16627648(97.26%)	9377091(56.39%)
ARoe_Ct_Inp pair	17231957	16419118(95.28%)	14881463(90.63%)
ARoe_Ct_Inp read1	17231957	17231840(100.00%)	15101273(87.64%)
ARoe_Ct_Inp read2	17231957	17231825(100.00%)	15017251(87.15%)
LacZ_Ct_AR pair	17940107	11971242(66.73%)	9630713(80.45%)
LacZ_Ct_AR read1	17940107	17902968(99.79%)	12174428(68.00%)
LacZ_Ct_AR read2	17940107	17902844(99.79%)	11648228(65.06%)
LacZ_D2d_AR pair	16409040	11534212(70.29%)	9315365(80.76%)
LacZ_D2d_AR read1	16409040	16027715(97.68%)	10681605(66.64%)
LacZ_D2d_AR read2	16409040	16024886(97.66%)	10422064(65.04%)
LacZ_D2d_Inp pair	24733178	23370827(94.49%)	21227560(90.83%)
LacZ_D2d_Inp read1	24733178	24732809(100.00%)	21586952(87.28%)

LacZ_D2d_Inp read2 24733178 24732818(100.00%) 21420590(86.61%)

Sample	Legnth of reads
ARoe_Ct_AR	176
ARoe_Ct_Inp	161
LacZ_Ct_AR	173
LacZ_Ct_Inp	163
LacZ_D2d_AR	192
LacZ_D2d_Inp	170

Sequencing depth

14510254

Antibodies

5ug of anti-Androgen Receptor antibody (PG-21, Cat. No. 06-680, Sigma)

Peak calling parameters

Peak calling was performed at the Novogene company facility, as follows: FASTA files were aligned using the Burrows-Wheeler Aligner (BWA) 70, and MACS2 software was used to identify narrow peaks with a q-value cut-off of 0.05.Peaks were annotated and merged using the annotatePeaks.pl and mergepeaks.pl functions from the HOMER software.

Data quality

q-value cut-off of 0.05 was used, and the following number of peaks per condition was obtained:
Experiment IP Input/Mock: Count of narrow peaks
AR_ARoe_Ct : 64872
AR_lacZ_Ct : 11417
AR_lacZ_D2d: 50992

Software

Sequencing was performed at the Novegene facility using the Novaseq 6000 platform. The original raw data from Illumina platform are transformed to Sequenced Reads, known as Raw Data or RAW Reads, by base calling of CASAVA. Raw data are recorded in a FASTQ file. The reads were mapped to the reference genome using BWA (Li, H. and R. Durbin, 2009).
By utilizing MACS2 software (Yong Zhang, Tao Liu et al., 2008) (threshold q value = 0.05) to finish the peak calling, we can calculate the number of peaks, the peak width and its distribution, and find the peak related genes.
